# Distribution and genetic characterization of fluoroquinolone resistance gene *qnr* among *Salmonella* strains from chicken in China

**DOI:** 10.1128/spectrum.03000-23

**Published:** 2024-02-27

**Authors:** Yang Chen, Lihui Liu, Yali Guo, Jinhua Chu, Bangjuan Wang, Yuxin Sui, Hanqi Wei, Haihong Hao, Lingli Huang, Guyue Cheng

**Affiliations:** 1 National Reference Laboratory of Veterinary Drug Residues (HZAU) and MAO Key Laboratory for Detection of Veterinary Drug Residues, Huazhong Agricultural University, Wuhan, Hubei, China; 2 MOA Laboratory for Risk Assessment of Quality and Safety of Livestock and Poultry Products, Huazhong Agricultural University, Wuhan, Hubei, China; 3 College of Veterinary Medicine, Huazhong Agricultural University, Wuhan, Hubei, China; Agriculture and Agri-Food Canada, Canada

**Keywords:** *qnr* genes, *Salmonella*, chicken production chain, genetic characteristics

## Abstract

The prevalence and dissemination of the plasmid-mediated fluoroquinolone (FQ) resistance gene *qnr* in *Salmonella* are considered serious public health concerns worldwide. So far, no comprehensive large-scale studies have focused on the prevalence and genetic characteristics of the *qnr* gene in *Salmonella* isolated from chickens. Herein, this study aimed to investigate the prevalence, antimicrobial resistance (AMR) patterns, and molecular characteristics of chicken-originated *qnr*-positive *Salmonella* strains from chicken farms, slaughterhouses, and markets in 12 provinces of China in 2020–2021. The overall prevalence of the *qnr* gene was 21.13% (56/265), with the highest prevalence in markets (36.11%, 26/72), followed in farms (17.95%, 21/117), and slaughterhouses (10.53%, 9/76). Only the *qnrS* and *qnrB* genes were detected, and the prevalence rate of the *qnrS* gene (19.25%, 51/265) was higher than that of the *qnrB* gene (1.89%, 5/265). Whole genome sequencing identified 37 distinct AMR genes and 15 plasmid replicons, and the most frequent mutation in quinolone resistance determining regions was *parC* (T57S; 91.49%, 43/47). Meanwhile, four different *qnrS* and two *qnrB* genetic environments were discovered among 47 *qnr*-positive *Salmonella* strains. In total, 21.28% (10/47) of the strains were capable of conjugative transfer, and all were *qnrS1*-positive strains, with the majority of transferable plasmids being IncHI2 types (*n* = 4). Overall, the prevalence of *qnr*-positive *Salmonella* strains from chickens in China and their carriage of multiple resistance and virulence genes and transferable plasmids is a major concern, which calls for continuous surveillance of *qnr*-positive *Salmonella* and the development of measures to control its prevalence and transmission.

**IMPORTANCE:**

*Salmonella* is a common foodborne pathogen responsible for 155,000 deaths annually worldwide. Fluoroquinolones (FQs) are used as first-line drugs for the treatment of *Salmonella* infections in several countries and regions. However, the emergence and increasing prevalence of the FQ-resistant gene *qnr* in *Salmonella* isolated from chickens have been widely reported. Gaining insight into the genetic mechanisms of AMR genes in chicken could lead to the development of preventive measures to control and reduce the risk of drug resistance. In this study, we identified *qnr*-positive *Salmonellae* isolated from chickens in different regions of China and their AMR patterns and genome-wide characteristics, providing a theoretical basis for further control of their prevalence and transmission.

## INTRODUCTION

*Salmonella* is a common foodborne pathogen that causes gastroenteritis in humans and livestock worldwide (1). Globally, an estimated 93.8 million humans are infected with *Salmonella* each year, of which 80.3 million cases are foodborne (2). Foods of animal origin, especially chicken and its products, are considered to be the major reservoirs for many *Salmonella* serotypes (3, 4). *Salmonella* contamination in the chicken production chain may occur at any stage of breeding, slaughter, processing, transportation, and sale (5), suggesting that *Salmonella* is extremely difficult to eliminate completely and poses a significant health risk to humans.

FQs are commonly used first-line drugs for the treatment of intestinal infections caused by *Salmonella* in humans and animals (6, 7). Unfortunately, the misuse of FQs has resulted in bacteria resistant to these agents in both animals and patients (8, 9). These strains are frequently multidrug-resistant (MDR), causing heavy disease burdens and economic losses (10). The World Health Organization has designated FQs-resistant *Salmonella* as a high-priority pathogen to research and develop new antibiotics (11). It is crucial to characterize the resistance mechanisms of this pathogen in order to reduce its prevalence effectively.

Chromosomal resistance to FQs is mainly associated with overexpression of efflux pumps, decreased drug uptake capacity, and chromosomal mutations in the quinolone-resistance-determining region (QRDR) of DNA gyrase (*gyrA* and *gyrB*) and/or topoisomerase (*parC* and *parE*) genes. Plasmid-mediated quinolone resistance (PMQR) determinants include *qnr* (*qnrA*, *qnrB*, *qnrC*, *qnrD*, *qnrE*, *qnrS*, and *qnrVC*), *aac(6')-lb-cr*, *oqxAB*, and *qepA* genes, which could contribute to decreased susceptibility to FQs among bacteria via horizontal gene transfer (HGT) (12). Among these, the *qnr* gene encodes pentapeptide repeat proteins that block the binding of FQs to DNA gyrase and topoisomerase IV (12). In recent years, *qnr* genes have continued to attract widespread attention because they represent a class of prevalent PMQR genes in livestock with a broad overall distribution and increasing variants (12).

The expression of the *qnr* gene commonly mediates only low levels of FQs resistance, but it was found to reduce the bactericidal efficacy of ciprofloxacin (13, 14) and levofloxacin (13) significantly and further facilitate selection for mutants with increased levels of FQs resistance (15, 16). In addition, studies have discovered that the *qnr* genes and extended-spectrum β-lactams (ESBLs) resistance gene usually coexist on the same plasmid (17–20). Infections caused by *qnr*-positive strains carrying multiple antibiotic resistance genes (ARGs) may pose a significant threat to antimicrobial therapy (particularly empiric therapy) for human and animal diseases (21). Therefore, an assessment of the genetic characteristics and prevalence patterns of *Salmonella* strains carrying the *qnr* gene would be highly beneficial.

Currently, most studies have focused on determinants linked to FQs resistance-related PMQR and QRDR in *Salmonella* of food, human, and animal origin (22–24). Among these studies, a comprehensive assessment of *Salmonella* carrying the *qnr* gene was limited by small sample sizes and incomplete detection of *qnr* alleles. Therefore, this study investigated resistance profiles and the molecular characterization of *qnr*-positive *Salmonella* isolated from chicken farms, slaughterhouses, and markets in Hunan, Hubei, Jiangxi, Anhui, Guangdong, Guangxi, Fujian, Hainan, Hebei, Shaanxi, Ningxia, and Xinjiang provinces. These data provide a theoretical basis for reducing the prevalence of the *qnr* gene in *Salmonella* from chicken in China.

## RESULTS

### Prevalence of *qnr*-positive *Salmonella* strains in the chicken production chain

**Fig 1:**
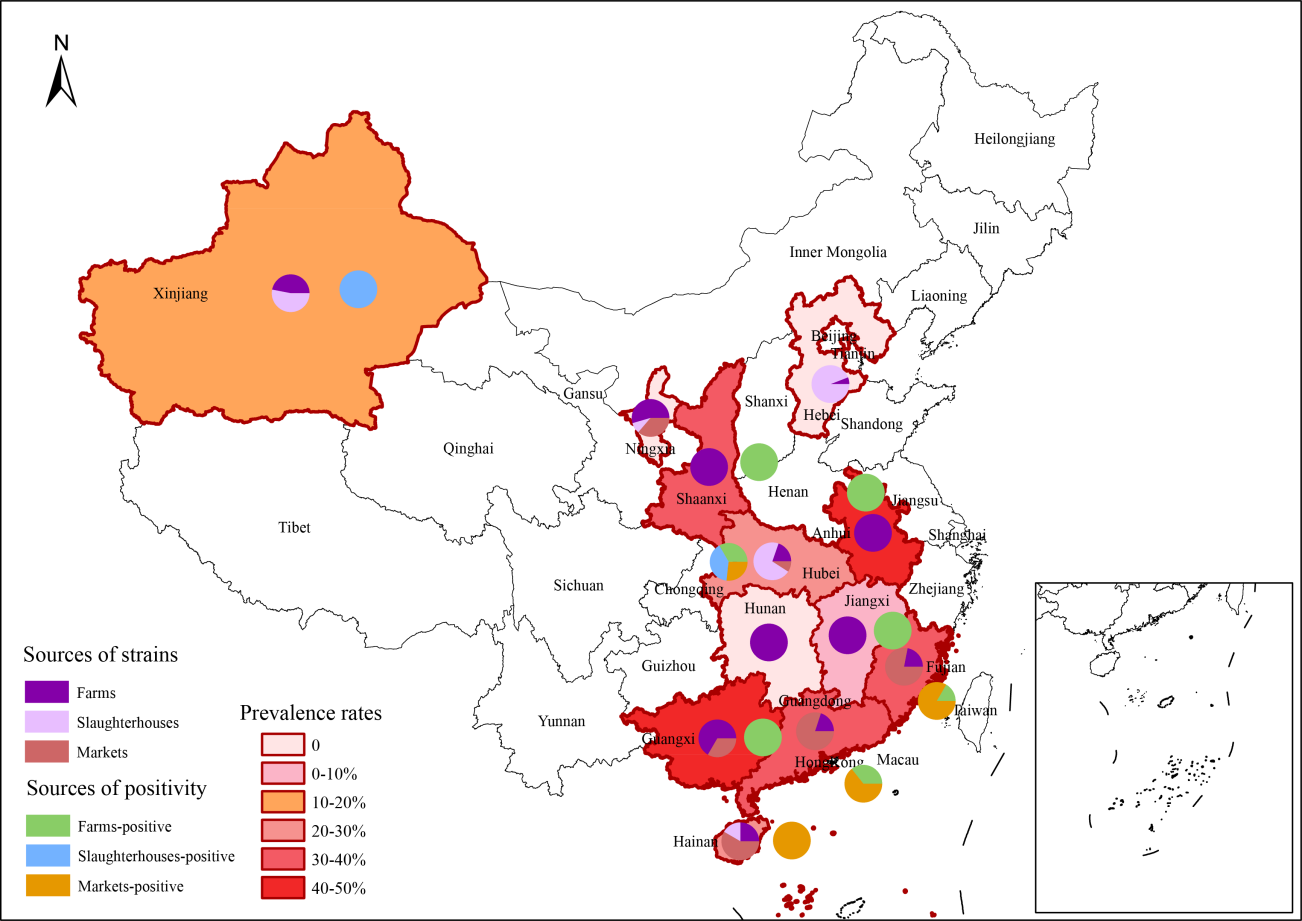
Map of the distribution of *qnr* genes in *Salmonella* collected from chicken production chain in China. The shade of color of the province represents the prevalence of *qnr* genes. Each province included the proportion of sources of strains and the positivity of *qnr* genes.

Among the 265 *Salmonella* strains collected from chicken farms, markets, and slaughterhouses in 12 provinces in China from 2020 to 2021, 56 *qnr*-positive strains were detected, representing an overall prevalence rate of 21.13% (Table 1). The prevalence of *qnr*-positive strains was different among different regions, with relatively high prevalence rates in Guangdong (40%, 14/35), Fujian (37.5%, 12/32), and Shaanxi (33.33%, 5/15), but none were detected in Hunan, Hebei, and Ningxia (Fig. 1). The prevalence of *qnr*-positive strains in farms, slaughterhouses, and markets was 17.95% (21/117), 10.53% (9/76), and 36.11% (26/72), respectively. Notably, the low number of samples may affect prevalence in some regions. A total of two *qnr* alleles were detected, including the *qnrS* gene (19.25%, 51/265) and the *qnrB* gene (1.89%, 5/265), whereas the *qnrA*, *qnrC*, *qnrD*, *qnrE*, and *qnrVC* genes were not detected. Of these, 51 *qnrS*-positive strains were distributed in 20 farms, 9 slaughterhouses, and 22 markets, and 5 *qnrB*-positive strains were distributed in 2 farms and 3 markets. Moreover, two variant *qnrS* genes (51 *qnrS1* and *1 qnrS2*) were identified, and all *qnrB* genes belonged to the *qnrB6* variant. One (GD-109) strain simultaneously carried the *qnrS1*/*S2* genes.

**TABLE 1:** Number of *Salmonella* (*n* = 265) from chicken farms, slaughterhouses, and markets in 12 provinces of China in 2020–2021^*a*^

Province	Source of strains			Positive rate of *qnr*
	Farms (*n* = 117)	Slaughterhouses (*n* = 76)	Markets (*n* = 72)	
Anhui	2 (1)	-	-	50% (1/2)
Hunan	36 (0)	-	-	0% (0/36)
Hubei	13 (5)	47 (6)	6 (4)	22.73% (15/66)
Jiangxi	15 (1)	-	-	6.67% (1/15)
Guangdong	7 (5)	-	28 (9)	40% (14/35)
Guangxi	4 (3)	-	2 (0)	50% (3/6)
Fujian	7 (2)	-	25 (10)	37.5% (12/32)
Hainan	3 (0)	2 (0)	7 (3)	25% (3/12)
Hebei	1 (0)	16 (0)	-	0% (0/17)
Shaanxi	15 (4)	-	-	33.33% (5/15)
Ningxia	6 (0)	1 (0)	4 (0)	0% (0/11)
Xinjiang	8 (0)	10 (3)	-	11.76% (3/18)
Positive rate of *qnr*	17.95% (21/117)	10.53% (9/76)	36.11% (26/72)	21.13% (56/265)

^*a*^ The number of positive strains belonging to the provinces is in brackets. The “-” indicates that the sample was not collected at this stage.

### Antimicrobial susceptibility of *qnr*-positive *Salmonella*

**Fig 2:**
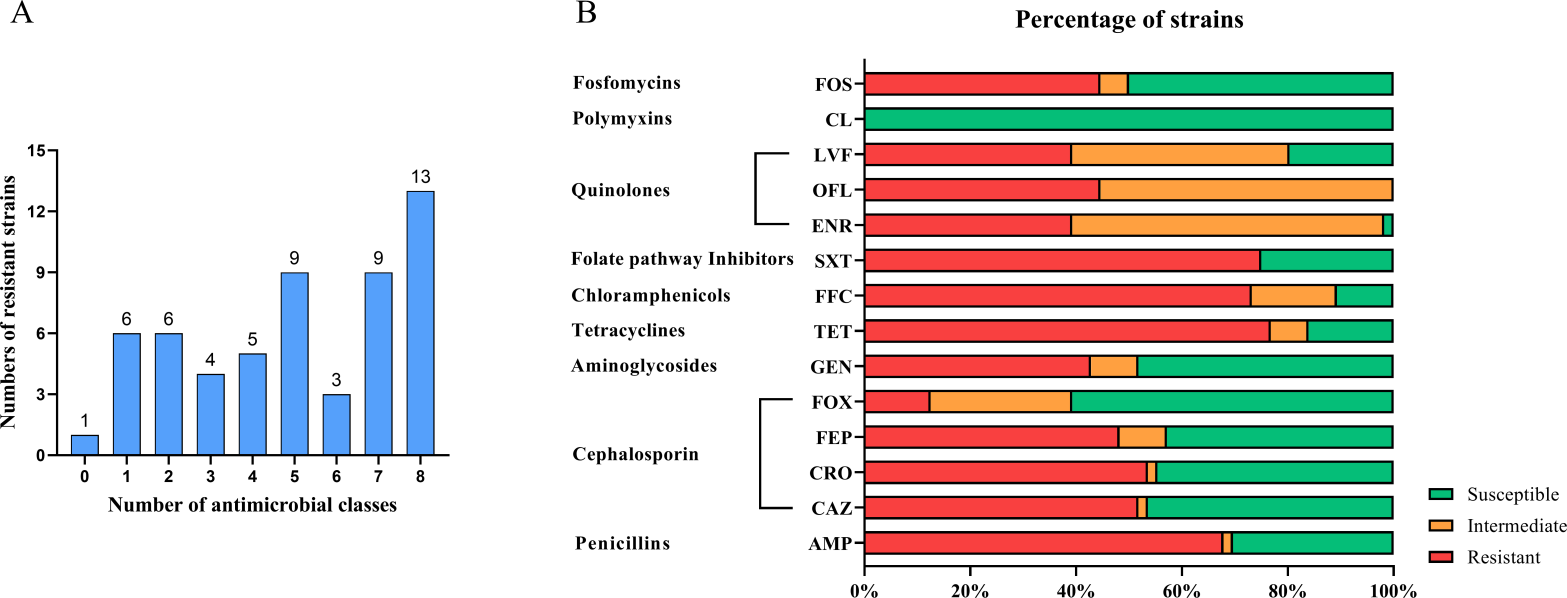
Antimicrobial resistance in *qnr*-positive *Salmonella* from chicken in China. (**A**) Numbers of *qnr*-positive *Salmonella* strains resistant to different antimicrobial classes. (**B**) Percentage of *qnr*-positive *Salmonella* strains resistant to different antimicrobial agents. Antimicrobial agent abbreviations: ampicillin (AMP), ceftazidime (CAZ), ceftriaxone (CRO), cefepime (FEP), cefoxitin (FOX), gentamicin (GEN), tetracycline (TET), florfenicol (FFC), trimethoprim-sulfamethoxazole (SXT), enrofloxacin (ENR), ofloxacin (OFL), levofloxacin (LVF), colistin (CL), and fosfomycin (FOS).

All 56 *qnr*-positive *Salmonella* strains in the present study were susceptible to colistin; one strain was susceptible to all classes of antimicrobials. A total of 76.79% (43/56) of strains were resistant to three or more antimicrobial classes (Fig. 2A). The rates of strains resistant to enrofloxacin, ofloxacin, and levofloxacin were 39.29% (22/56), 44.64% (25/56), and 39.29% (22/56), respectively, and 35.09% (20/56) of strains were resistant to all FQs tested. In addition, the majority of the strains were resistant to tetracycline (76.79%, 43/56), followed by trimethoprim-sulfamethoxazole (75.00%, 42/56), florfenicol (73.21%, 41/56), ampicillin (67.86%, 38/56), ceftriaxone (53.57%, 30/56), and ceftazidime (51.79%, 29/56) (Fig. 2B). Relatively low resistance rates were observed for cefoxitin (10.53%, 6/56).

### Serotype, multilocus sequence type, and phylogenesis

**Fig 3:**
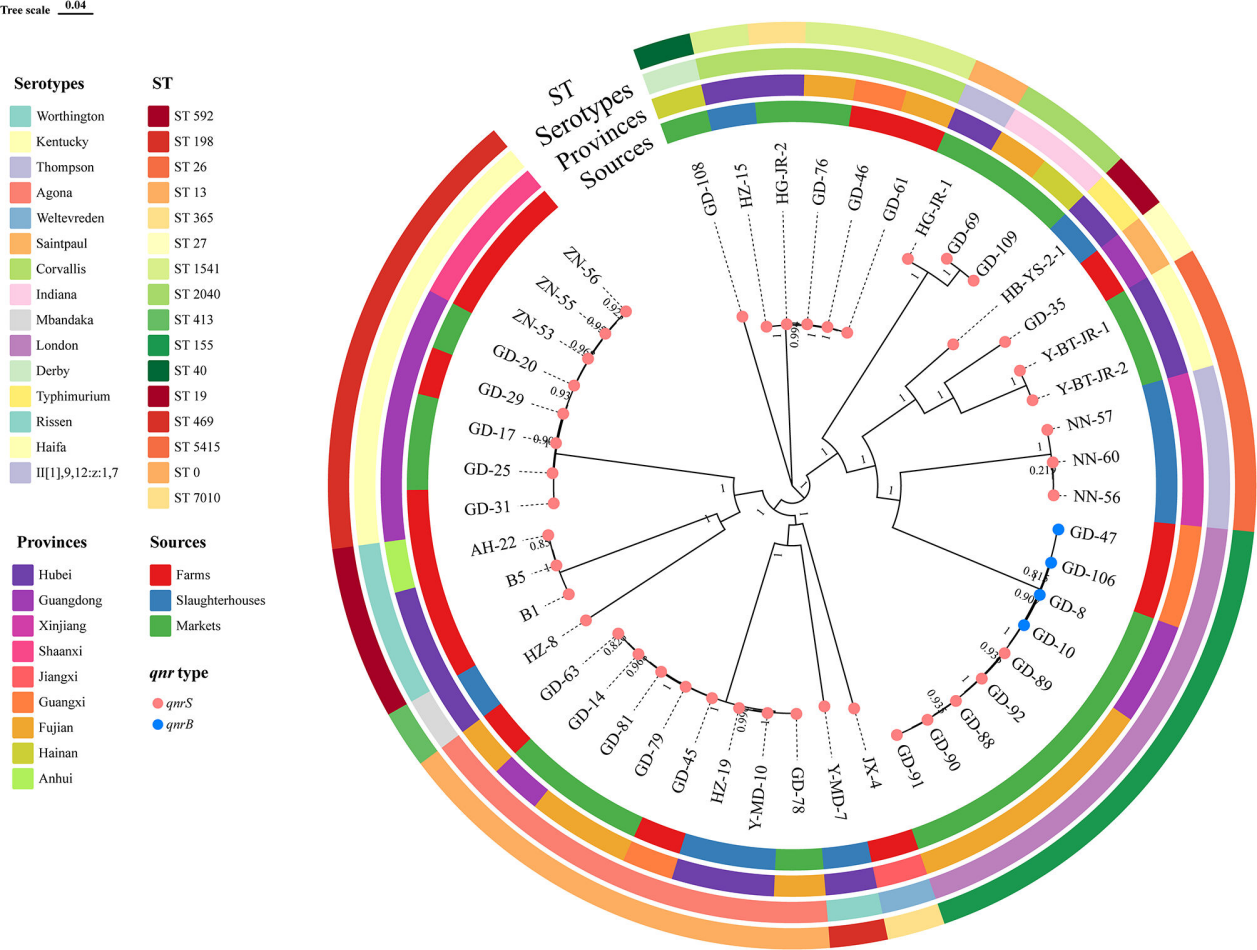
Phylogenetic diversity, sources, provinces, serotypes, and multilocus sequence types (MLST) in *qnr*-positive *Salmonella*. The ST 0 represented an unknown MLST type.

Fifteen serotypes were identified in 47 nonclonal *qnr*-positive *Salmonella* strains using the SeqSero2 database (Fig. 3). London (*n*  =  9, 19.15%) was the most prevalent serotype, followed by Agona and Kentucky (*n*  =  8, 17.02%), Corvallis (*n*  =  5, 10.87%), Worthington and Thompson (*n*  =  3, 6.52%), and Indiana and Haifa (*n*  =  2, 4.35%). Only one isolate was obtained for Weltevreden, Mbandaka, Rissen, Saintpaul, Typhimurium, Derby, and II[1],9,12:z:1,7. Notably, all four strains carrying *qnrB* were serotype London.

Fifteen distinct ST types were recognized using the multilocus sequence type (MLST) database, except for one (II[1],9,12:z:1,7) that has an unknown ST. The ST 115 (*n*  =  9), ST 13 (*n*  =  8), ST 198 (*n*  =  8), ST 592 (*n*  =  3), ST 2040 (*n*  =  2), ST 5415 (*n*  =  2), and ST 26 (*n*  =  2) were assigned one-to-one correspondence to serotypes London, Agona, Kentucky, Worthington, Indiana, Haifa, and Thompson, respectively (Fig. 3). In addition, the Corvallis serotype had two ST types, including ST 1541 (*n*  =  4) and ST 7010 (*n*  =  1). For individual *Salmonella* serotypes, ST 365 (Weltevreden), ST 413 (Mbandaka), ST 469 (Rissen), ST 27 (Saintpaul), ST 19 (Typhimurium), ST 7010 (Corvallis), and ST 40 (Derby) were identified.

Single nucleotide polymorphism (SNP) phylogenetic analysis using the genome of *Salmonella* Typhimurium LT2 (RefSeq accession number NC003197.2) as the reference revealed that all strains covered 76.70% of the reference genome, with 3,797,871 nucleotide positions present in all analyzed genomes. The SNP phylogenetic analysis was performed to evaluate the relationship among the 47 *qnr*-positive *Salmonella* strains, and the result revealed that the strains were clustered into respective serovars, independently of the origin of the strains (Fig. 3).

### Antimicrobial, biocide, and metal resistance genes

**Fig 4:**
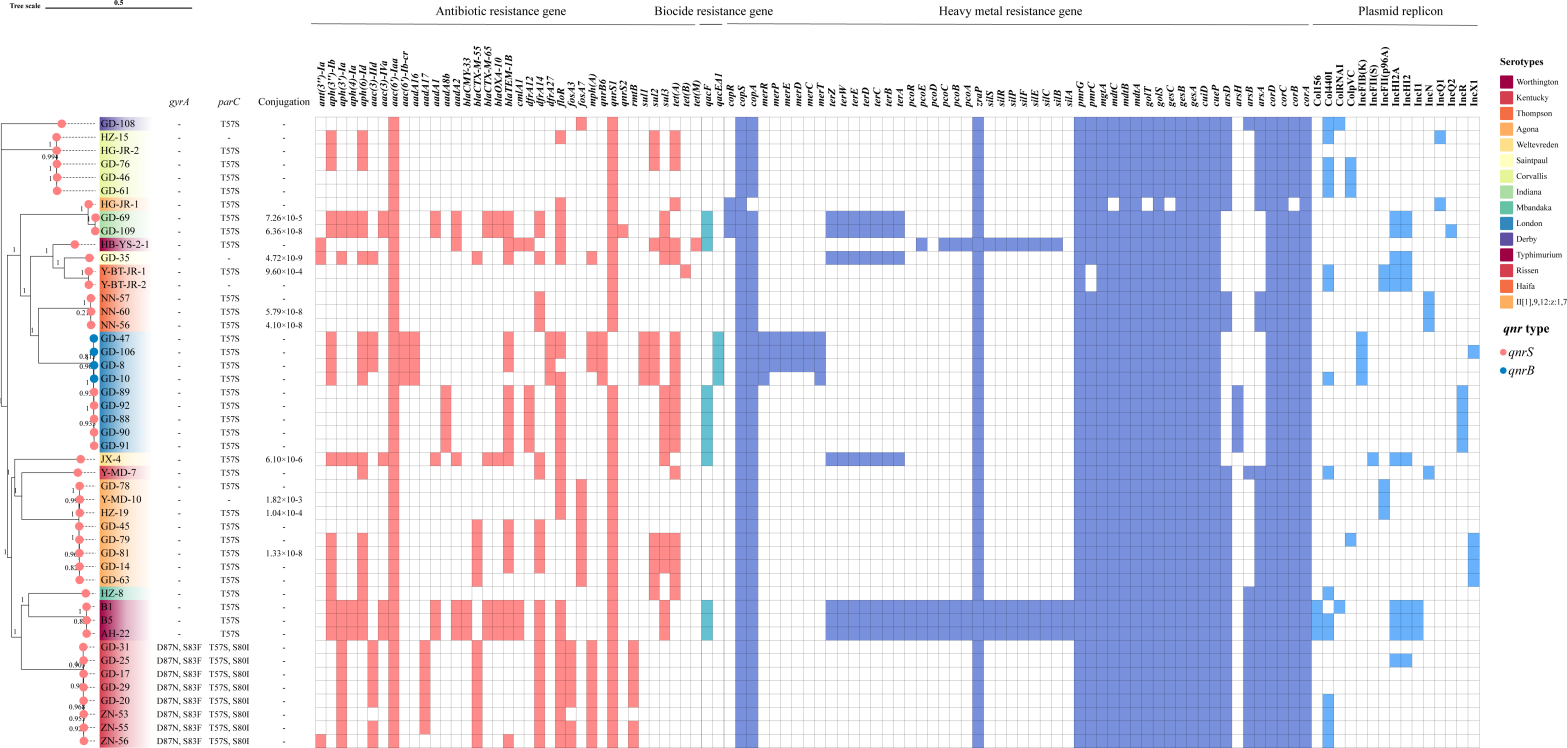
Phylogenetic diversity, mutations in QRDR of *gyrA* and *parC* genes, conjugation frequencies, antibiotic, biocide, and metal resistance genes, and plasmid replicons in *qnr*-positive *Salmonella*.

Thirty-seven distinct ARGs were identified among 47 non-clonal *qnr*-positive *Salmonella* strains using the Resfinder database (Fig. 4). *aac(6′)-Iaa* encoding resistance to aminoglycosides was detected in all strains (100%). Thirty (63.83%) and 23 (48.94%) strains carried the florfenicol-resistant gene *floR* and the sulfonamide-resistant gene *dfrA14*, respectively. Notably, ~45% of the strains carried the *bla_TEM-1B_* and *tet*(*A*) genes, which encode resistance to β-lactams and tetracyclines. Six β-lactam resistance genes (*bla_TEM-1B_, bla_CTX-M-55_, bla_CTX-M-65_, bla_OXA-10_, bla_LAP-2_*, and *bla_CMY-33_*) were identified in 29 (61.7%) strains. The most commonly detected β-lactam resistance gene was *bla_TEM-1B_* (44.68%, 21/47), followed by *bla_CTX-M-55_* (29.79%, 14/47), *bla_CTX-M-65_*, and *bla_OXA-10_* (12.77%, 6/47). 25.53% (12/47) of strains carried two or more β-lactam resistance genes, and the most common combination was *bla_TEM-1B_* and *bla_CTX-M-55_* (*n*  =  6). A total of 43 strains were observed with missense mutations in QRDR of the *gyrA* and/or *parC* genes, while the *gyrB* and *parE* genes had no missense mutations (Fig. 4). Thirty-five strains with only mutations in the *parC* (T57S) gene showed 18 strains of FQs-resistant and 17 strains of susceptible phenotypes, respectively. Eight strains that contained both mutations in the *gyrA* (D87N and S83F) and *parC* (T57S and S80I) genes exhibited a high level of FQs resistance, all belonging to the Kentucky serotype. Overall, resistance phenotypes and ARGs were highly correlated (Table 2).

**TABLE 2:** Evaluation of genotype prediction of phenotypic resistance

Antimicrobial	Phenotypic resistant (*n*)		Phenotypic susceptible (*n*)		Sensitivity^*b*^ (%)	Specificity^*c*^ (%)
	Genotypic resistant (TP)^*a*^	Genotypic susceptible (FN)^*a*^	Genotypic resistant (FP)^*a*^	Genotypic susceptible (TN)^*a*^		
AMP	30	1	0	16	96.77	100.00
CAZ	21	1	0	25	95.45	100.00
CRO	21	2	0	24	91.30	100.00
FEP	18	1	2	27	94.74	91.30
FOX	3	1	0	43	75.00	100.00
ENR	17	0	30	0	100.00	NA^*d*^
OFL	20	0	27	0	100.00	NA^*d*^
LVF	17	0	30	0	100.00	NA^*d*^
GEN	16	0	0	31	100.00	100.00
TET	22	12	0	13	64.71	100.00
FFC	30	2	0	15	93.75	100.00
SXT	32	0	1	14	100.00	93.33
FOS	20	4	0	23	83.33	100.00
Total	267	24	90	231	91.75	71.96

^*a*^ TP, true positive; FN, false negative; FP, false positive; TN, true negative.

^*b*^ Sensitivity = TP/(TP + FN).

^*c*^ Specificity = TN/(TN + FP).

^*d*^ NA represents sensitivity could not be calculated because each strain contains at least one fluoroquinolone resistance gene. Antimicrobial agent abbreviations: ampicillin (AMP), ceftazidime (CAZ), ceftriaxone (CRO), cefepime (FEP), enrofloxacin (ENR), ofloxacin (OFL), levofloxacin (LVF), gentamicin (GEN), tetracycline (TET), florfenicol (FFC), trimethoprim/sulfamethoxazole (SXT), and fosfomycin (FOS).

The biocide and metal resistance genes were predicted based on the BacMet database (Fig. 4). The biocide resistance genes *qacEΔ1* and *qacF* were detected in 4 (8.51%) and 12 (25.53%) strains, respectively. Notably, all *qnr*-positive *Salmonella* strains harbored the chromosome-associated heavy metal resistance genes: the gold resistance genes *gesAB* and *golS*, the magnesium resistance gene *mgtA*, the copper resistance genes *cueP*, *cuiD*, and *copS*, the cobalt resistance genes *corACD*, the zinc resistance genes *mdtAB* and *zraP*, and the iron resistance gene *pmrG*. In addition, plasmid-associated genes for resistance to arsenic (*arsA*, *arsB*, *arsH*, and *arsD*), silver (*silA*, *silB*, *silC*, *silE*, *silF*, *silP*, *silR*, and *silS*), copper (*copA*, *copR*, *pcoA*, *pcoB*, *pcoC*, *pcoD*, *pcoE*, and *pcoR*), tellurium (*terA*, *terB*, *terC*, *terD*, *terE*, *terW*, and *terZ*), and mercury (*merT*, *merC*, *merD*, *merE*, *merP*, and *merR*) were also detected.

In total, 41 (87.23%) strains carried at least one plasmid replicon, 19 (40.43%) carried two or more, and 7 (14.89%) carried three or more. The most commonly detected plasmid was Col440I (34.04%, 16/47), followed by IncHI2 and IncHI2A (14.89%, 10/47).

### Virulence genes

**Fig 5:**
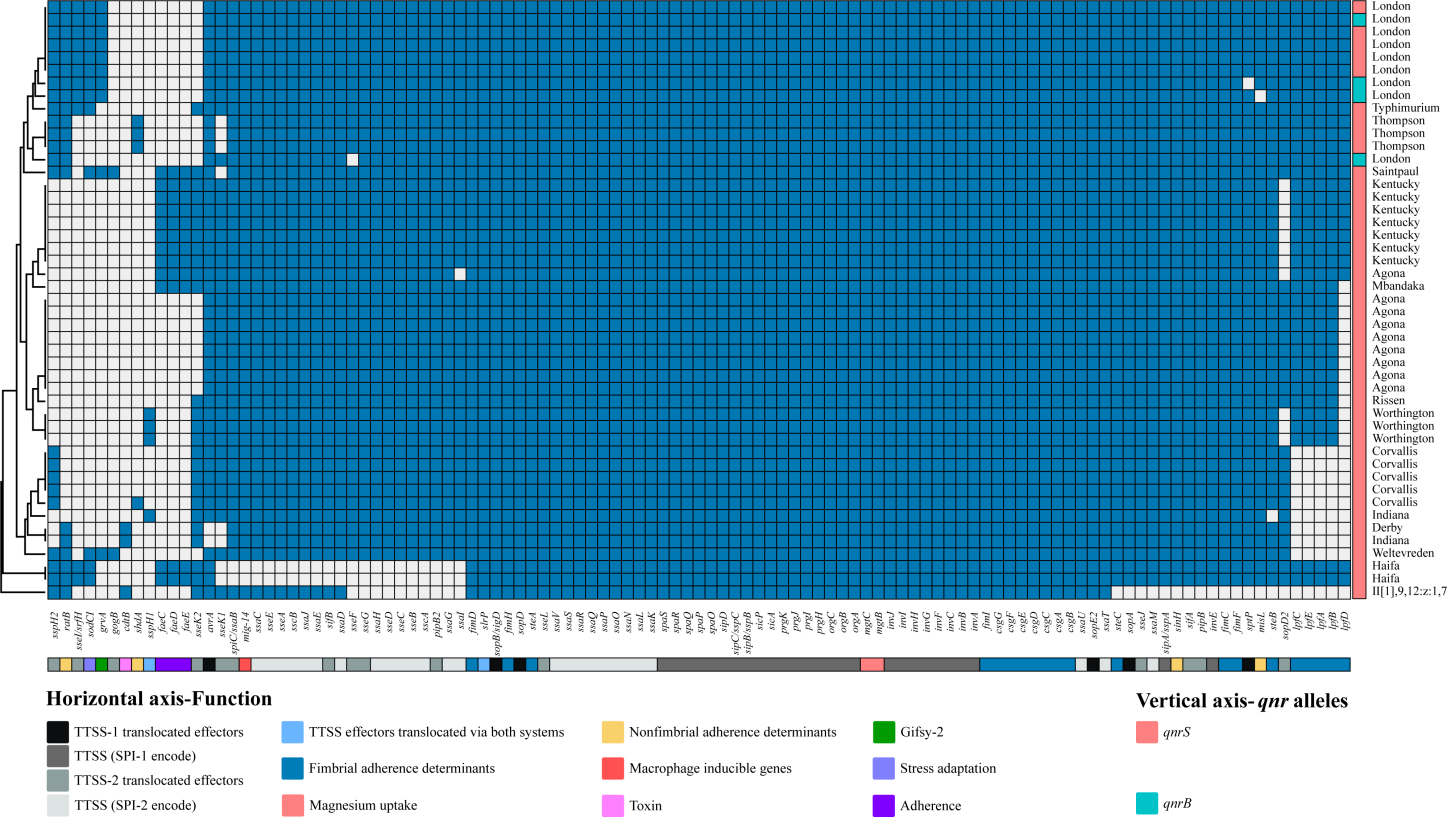
Dendrogram of the hierarchical clustering heat map of *qnr*-positive *Salmonella* and virulence genes. The figure shows the prediction of virulence gene factor profiles for the studied isolates. The dendrogram (unweighted pairwise grouping method using arithmetic averaging) was based on pairwise similarity relationships according to the presence or absence of individual virulence genes. The vertical axis is the isolate number and the horizontal axis is the selected virulence genes identified. Blue cells, presence of gene; white cells, absence of gene.

A total of 109 virulence genes were detected among 47 *qnr*-positive *Salmonella* strains (Fig. 5). Our results showed that the distribution of virulence genes was closely correlated with serotypes. These virulence genes were classified as fimbrial adherence determinants, macrophage inducible genes, magnesium uptake, nonfimbrial adherence determinants, secretion system, adherence, stress adaptation, gifsy-2, and toxin. It was shown that the distribution of virulence genes was closely associated with serotype. A total of 11 *Salmonella* pathogenicity islands (SPI-1, SPI-2, SPI-3, SPI-4, SPI-5, SPI-8, SPI-9, SPI-13, SPI-14, C63PI, and CS54_island) were detected in 47 *qnr*-positive *Salmonella* strains. Of these, all strains carried the typical virulence factors of SPI-1 and SPI-2 and the *mgtCB* genes of SPI-3. However, plasmid-borne genes *spv* were not detected in our study. Using PathogenFinder, 630–1,145 pathogenic families were predicted matching to the genomes of 47 *qnr*-positive *Salmonella* strains with a probability of 92.9%–94.5% as human pathogens, indicating all *qnr*-positive *Salmonella* strains were probably pathogenic to humans.

### Genetic environment of *qnr* genes

**Fig 6:**
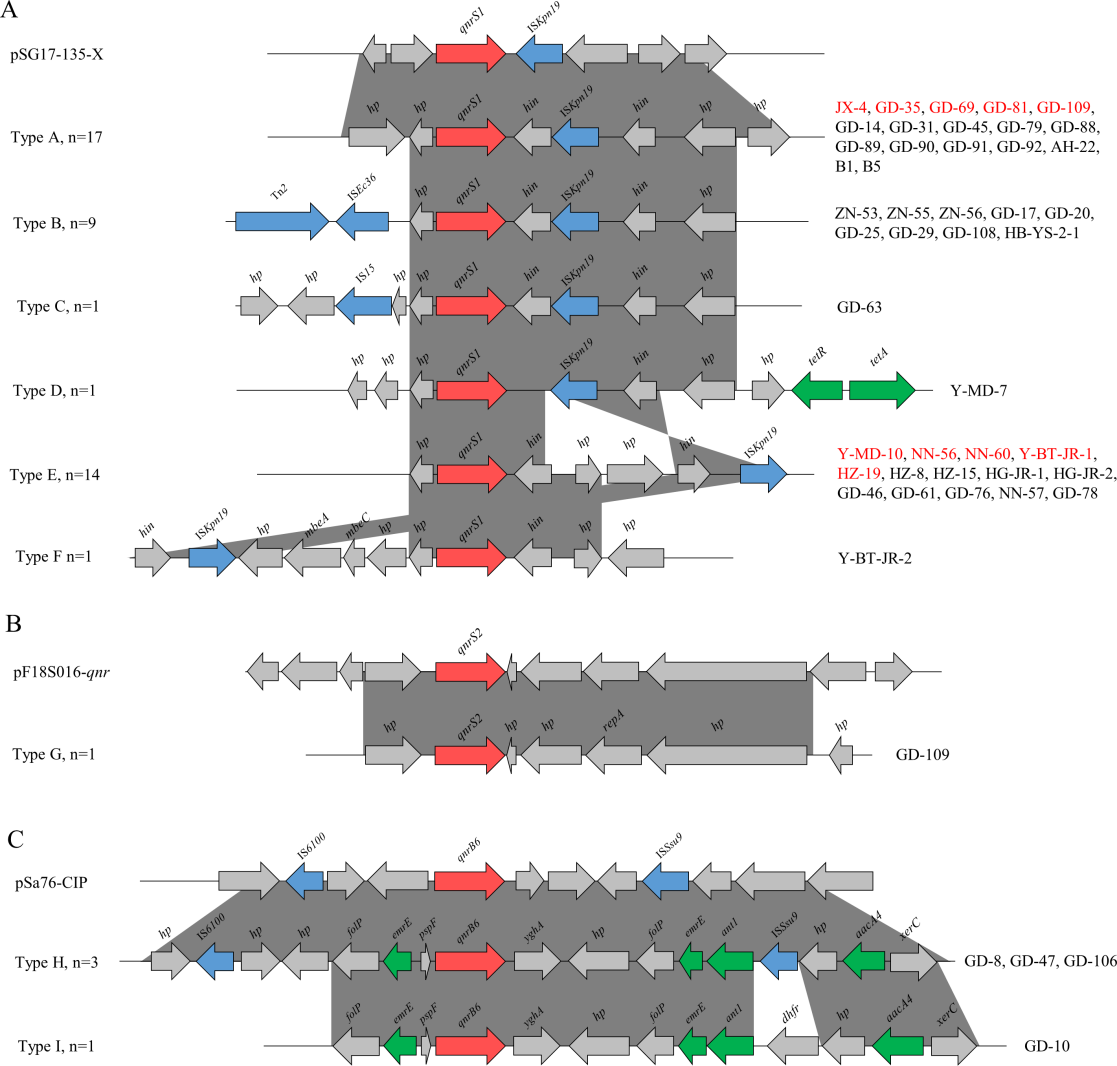
The genetic environment of *qnr* genes in 47 *qnr*-positive *Salmonella* strains. (**A**) Genetic environment in close proximity to *qnrS1*. pSG17-135-X (NCBI ref. CP048777.1) was the reference genetic structure. (**B**) Genetic environment in close proximity to *qnrS2*. pF18S016-qnr (NCBI ref. CP082903.1) was the reference genetic structure. (**C**) Genetic environment in close proximity to *qnrB6*. pSa76-CIP (NCBI ref. NZ_MG874044.1) was the reference genetic structure. *qnr* genes, mobile genetic elements, and other ARGs were displayed with red, blue, and green arrows, respectively. *hp* represented gene encoding hypothetical protein. Strains in red represented capable of conjugative transfer.

Genetic environment analyses revealed that 45 strains (97.83%) contained at least one mobile genetic element (MGE) near the *qnr* gene based on short-read sequencing of 47 *qnr*-positive *Salmonella* strains. The result revealed various and complex genetic environments of *qnr*-positive *Salmonella* during the transmission of the *qnr* gene. As shown in (Fig. 6A), *qnrS1* was most frequently found in the *qnrS1*-IS*Kpn19* cluster (*n*  =  33; type A, *n*  =  17; type D, *n*  =  1; type E, *n*  =  14; type F, *n*  =  1), followed by the Tn*2*-IS*Ec36-qnrS1*-IS*Kpn19* cluster (type B, *n*  =  9), and the IS*15-qnrS1-*IS*Kpn19* cluster (type C, *n*  =  1). The *qnrS1* downstream regions of type B and C were Tn2-IS*Ec36* and IS*15*, respectively, which is the main difference between the *qnrS1*-IS*Kpn19* clusters. We found that the IS*Kpn19* gene was closely linked to the DNA convertase gene *hin* and was present in all genetic environments of *qnrS1*. However, MGE was not detected in the genetic environment structure of *qnrS2* (Fig. 6B). The *qnrB* gene was found in the IS*Ssu9-qnrB6*-IS*6100* cluster (*n*  =  3), while no MGEs were found in the one remaining strain (Fig. 6C).

### Transmissibility

**Fig 7:**
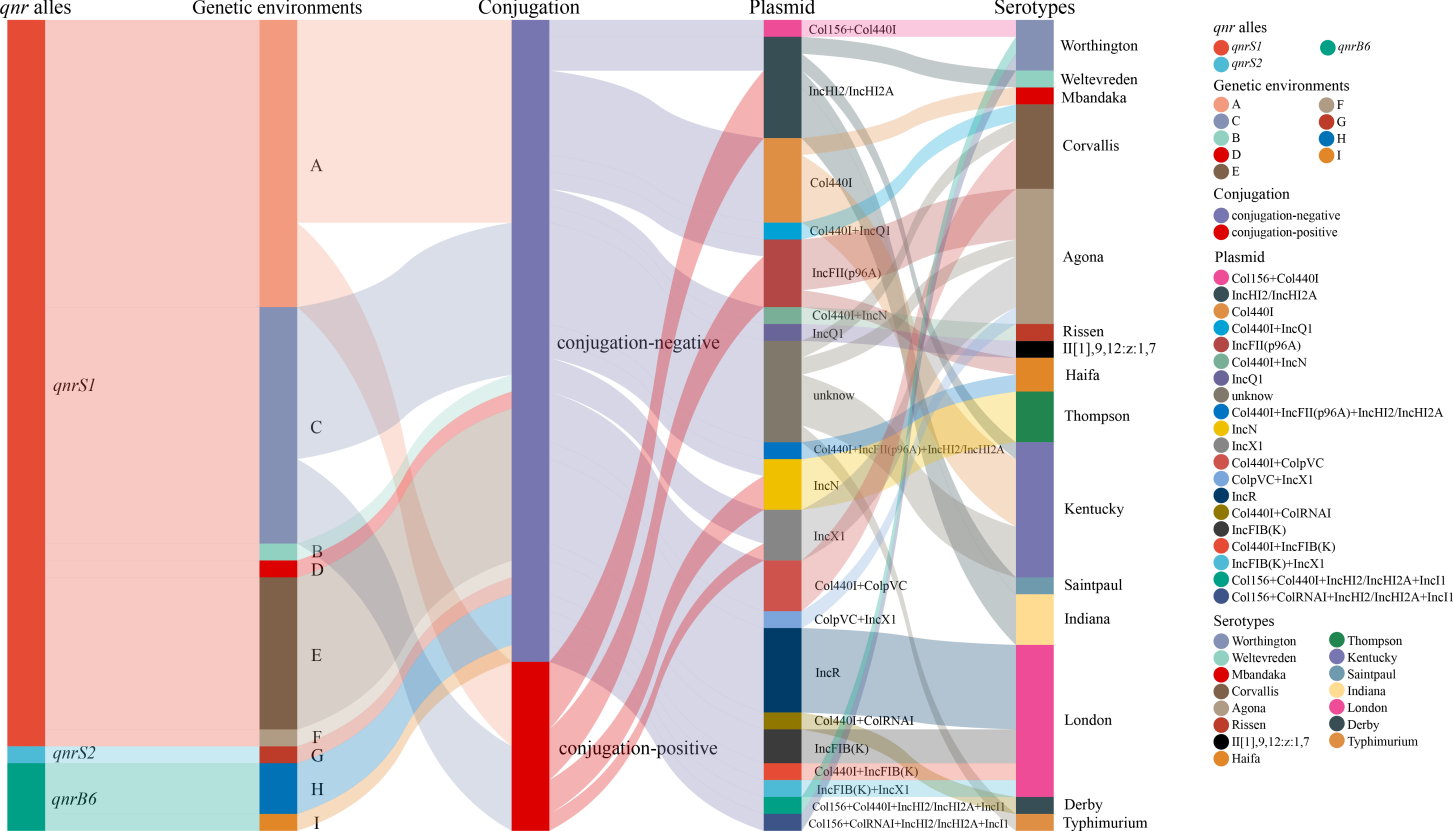
Sankey diagram combining the *qnr* types, genetic environments of *qnr* gene, transferability, plasmid replicons, and serotypes.

To assess the transferability of plasmids carrying the *qnr* gene, conjugation assays were performed for the 47 *qnr*-positive strains with *Escherichia coli* J53. The results showed that 21.27% (10/47) of the strains were transferable, and all were *qnrS1*-positive strains, with conjugation frequencies ranging from 1.82 × 10^−3^ to 4.72 × 10^−9^ (Fig. 4 and 7). All *qnr*-positive strains were further analyzed by combined with genetic environments, transmissibility, plasmid types, and serotypes (Fig. 7). The plasmid types of the 10 transferable strains were mainly IncHI2 types (*n* = 4), followed by IncN and IncFII (p96A) (*n* = 2). Types A (*n* = 5) and C (*n* = 5) were the main genetic environments for transferable strains, and these strains were found in a variety of serotypes, mainly Agona (*n* = 3), followed by Thompson and Indiana (*n* = 2). Type A is predominantly present in the IncHI2 types (*n* = 4), and type C is present in the IncFII (p96A) and IncN types (*n* = 2).

## DISCUSSION

Food animals, particularly chickens, have long been recognized as important reservoirs for *Salmonella* (3). However, with the extensive abuse and misuse of antibiotics in the poultry industry, the prevalence of FQs-resistant *Salmonella* has increased in recent decades (12). Among these, the *qnr* gene, an essential molecular feature, has a potential risk of transmission to humans through the food chain, posing a serious threat to human health (24). In this study, we conducted a comprehensive molecular typing study of *qnr*-positive *Salmonella* isolated from chicken in China. The present study reported that the cumulative prevalence of *qnr*-positive *Salmonella* was 21.13% in 12 provinces in China, which was lower than those reported for similar poultry production systems in other districts of China, such as Shanghai (71.23%) (25), Hangzhou (24.59%) (26), and Guangzhou, Shandong, and Zhejiang (46.5%) (27), but higher than those reported in Sichuan (17.57%) (28). This indicated that the spread of the *qnr* gene in *Salmonella* isolated from chicken in China is relatively serious and that controlling the prevalence and spread of this gene should be a priority area for supervision. The United States, China, Brazil, and the European Union are the largest countries in poultry production (29), of which FQs are only banned for use in the United States. Meanwhile, the prevalence of the *qnr* gene in *Salmonella* strains was lower in North Carolina, the United States (0.24%) (5) relative to China (this study), Brazil (3.64%) (30), and the European Union (31), suggesting that the use of FQs may be closely related to the prevalence of the *qnr* gene. Several studies pointed out that the *qnrS* and *qnrB* genes were commonly detected in *Salmonella* isolated from chicken, together with the rare detection of other alleles, similar to our research (25, 28). Furthermore, the detection rate of the *qnrS* gene (19.25%) was higher than that of the *qnrB* gene (1.89%) in our study. The *qnrS* gene has gradually become dominant in *Salmonella* isolated from chicken in China in recent years (25, 27, 32, 33), possibly because plasmids carrying the *qnrS* gene may have a competitive advantage against selection pressure due to their low fitness cost relative to other alleles (34, 35). However, the patterns and related molecular mechanisms of the *qnrS* gene during transmission need to be further explored.

Our results also showed that 76.79% and 60.71% of *qnr*-positive *Salmonella* strains were resistant to three and five antimicrobial classes, respectively, which was higher than the 70.53% and 49.47% of chicken-derived *Salmonella* without detectable *qnr* genes (36). The majority of strains in our study were resistant to multiple antibacterials, meaning that *qnr*-positive *Salmonella* may be at greater risk for drug resistance and treatment failure. The majority of strains were resistant to tetracycline (74.07%), trimethoprim-sulfamethoxazole (72.22%), and ampicillin (70.37%), which are traditional first-line antibiotics used against bacterial infections in animal farms worldwide (37). The resistance to beta-lactams was also found in 63.83% (30/47) of the strains in this study, consistent with other reports that indicated high-level resistance to beta-lactams in *qnr*-positive *Salmonella* strains obtained from chicken (38, 39). In this study, the resistance rates of strains to enrofloxacin, ofloxacin, and levofloxacin were 42.59%, 46.30%, and 40.74%, respectively. Although *qnr* mediates only low levels of FQ resistance, its role in reducing susceptibility to FQs cannot be ignored.

Fifteen serotypes were detected in *qnr*-positive *Salmonella*, among which London, Agona, and Kentucky were the most common serotypes. The presence of *qnr*-positive plasmids in various *Salmonella* serotypes suggested that these plasmids have broad adaptability to different serotypes of *Salmonella*. The serotypes found in the chicken samples were similar to those identified in Guangdong, China (40) and Hanoi, Vietnam (41). Additionally, serovar Agona is recognized as one of the reasons for the increasing number of foodborne disease outbreaks (42); serovar London has been reported in various sources (patients, meat products, and the environment) (43, 44); and serovar Kentucky is one of the most common serovars isolated from broiler chickens in the United States (45), but is unlikely to cause human infection (46).

All *qnr*-positive *Salmonella* strains harbored the *aac(6′)-Iaa* gene. However, the *aac(6′)-Iaa* gene is usually transcriptionally silenced and rarely transcriptionally active and therefore does not confer resistance to aminoglycosides in *Salmonella* (47). Overall, 63.04% of strains co-existed with β-lactam resistance genes. Similarly, a high level of *qnr* genes in ESBL-producing *Salmonella* strains was observed in previous studies (48, 49). This suggests that the *qnr* gene may be closely linked to the β-lactam resistance genes in *Salmonella* and that cross-resistance to antibiotics is a particular concern. In our study, the ampicillin resistance *bla_TEM-1B_* gene was the most common β-lactam resistance gene, which is also the predominant beta-lactam gene in most *Salmonella* serotypes worldwide (33). Many strains were found to carry the *floR*, *dfrA14*, and *tet(A*) genes. Of note, it is quite common to have those genes in *Salmonella* (50, 51). Furthermore, we found that resistance genes to biocide (*qacEΔ1* and *qacF*) and heavy metals (gold, magnesium, copper, cobalt, zinc, iron, arsenic, silver, mercury, and tellurium) were common in *qnr*-positive *Salmonella* strains isolated from chicken. Numerous studies have shown that the widespread use of disinfectants and heavy metals may generate intense selection pressure, resulting in the emergence of cross-resistance and co-resistance (52, 53). Therefore, careful consideration needs to be given to the selection and use of disinfectants and heavy metal-containing feeds. For the first time, we identified the 16S rRNA methylase gene *rmtB1* in *qnr*-positive *Salmonella* strains that exhibited high levels of resistance to aminoglycoside. In our study, 82.98% (39/47) of *qnr*-positive *Salmonella* strains had no mutant (4/39) or T57S *parC* substitution (35/39), similar to those reported in Korea (23) and China (54). Notably, the T57S *parC* substitution is more of a naturally occurring compensatory mutation (55, 56) that is unrelated or doubtfully related to the FQs resistance phenotype (57). In addition, double missense mutations in *gyrA* (D87N and S83F) and *parC* (T57S and S80I) were detected in 17.02% (8/47) of strains, and these are considered the major target-site mutations (58). Although the mutation sites in *gyrA* and *parC* were similar to previous studies, the frequency of mutants in *qnr*-positive strains is much higher than in *qnr*-free strains (59). This indicated that the potential risk of mutations in *qnr*-positive *Salmonella* in QRDR may always be present. We found that *qnr* genes are located on multiple types of plasmids that spread across different regions and sources. In addition, 40% of the transferable plasmid types were IncHI2 and IncHI2A. Several studies have shown that IncHI2 and IncHI2A plasmids are major plasmid lineages contributing to the spread of antibiotic resistance in *Salmonella* (60, 61).

In our study, IS*Kpn19* was detected in all genetic environments of *qnrS1* regardless of the source, suggesting that IS*Kpn19* may play a pivotal role in the prevalence and spread of *qnrS1*. Flagellum phase variations are regulated by the Hin DNA invertase in pathogens to evade host immune responses (62). We found that the *hin* gene was linked with IS*Kpn19*, indicating a high transfer of the *hin* gene by IS*Kpn19*-directed HGT events, which would help the *qnr*-positive *Salmonella* to evade host immune responses. Furthermore, the *qnrS1* gene has been discovered to be related to several MGEs, such as Tn*3*-like transposons and insertion sequences IS*Ecl*, IS*26*, and IS*2*-like (63), which is similar to our results.

The virulence factors of *Salmonella* play an important role in adhesion, invasion, and multiplication of host cells. Our study detected typical virulence factors of SPI-1 and SPI-2 in all *qnr*-positive *Salmonella* strains. SPI-1 and SPI-2 in *Salmonella* serotypes contain highly conserved genes (64) that encode type III secretion systems (T3SS) responsible for invasion and intracellular replication (65). In addition, the *mgtC* locus on SPI-3 facilitates the adaptation of bacteria to the low Mg^2+^ and low pH endosomal environment resulting from SPI-1-mediated invasion (66). However, the plasmid-borne genes *spv* were not detected in *qnr*-positive *Salmonella* strains in our study. This may be due to the fact that AMR-virulence hybrid plasmids are commonly found in Typhimurium, Cholerasuis, Dublin, and Enteritidis serotypes (67), which were rarely detected in our study. We predicted the probability that the 47 *qnr*-positive *Salmonella* strains were human pathogens using PathogenFinder. Unexpectedly, all *qnr*-positive *Salmonella* strains were pathogenic, which could impose a heavy disease burden on humans. However, the pathogenic potential of *qnr*-positive *Salmonella* is required for further investigation.

### Conclusion

In this study, we demonstrated an accurate genetic characterization of the *qnr* gene in *Salmonella* isolated from chickens in China. We found that *qnr*-positive *Salmonella* strains were widely distributed in chicken from various regions of China, with a high prevalence of the *qnrS* gene. Importantly, we report a high prevalence of MDR *qnr*-positive *Salmonella* and the presence of a large number of virulence and resistance determinants that pose a serious risk to food safety. Meanwhile, we reported four different genetic environments for *qnrS* and two for *qnrB*. Therefore, it is essential to continue monitoring *qnr*-positive *Salmonella* and implement prevention and control strategies to reduce its prevalence and spread.

## MATERIALS AND METHODS

### Bacteria

A total of 265 *Salmonella* strains were isolated from the chicken farms, slaughterhouses, and markets in Hunan, Hubei, Jiangxi, Anhui, Guangdong, Guangxi, Fujian, Hainan, Hebei, Shaanxi, Ningxia, and Xinjiang provinces of China from 2020 to 2021. The strains were isolated from cloacal swabs of healthy chicken from farms (*n*  =  117), chicken cecal contents from slaughterhouses (*n*  =  76), and chicken meat and intestines obtained from markets (*n*  =  72).

### Identification of *qnr* genes

All *Salmonella* strains were screened for *qnrA*, *qnrB*, *qnrC*, *qnrD*, *qnrE*, *qnrS*, and *qnrVC* genes by PCR using primers as shown in Table 3 (68). The PCR conditions were as follows: one predenaturation cycle at 95°C for 5 min, 34 cycles of denaturation at 95°C for 30 s, annealing at *T*_m_ for 30 s, and elongation at 72°C for 30 s, and one postelongation cycle at 72°C for 5 min.

**TABLE 3:** PCR primers of *qnr* genes

Gene	Sequence (5′−3′)	Size (bp)	*T*_m_ (°C)	Reference
*qnrA*	F: ATTTCTCACGCCAGGATTTG R: GATCGGCAAAGGTTAGGTCA	515	53	(68)
*qnrB*	F: AGTCGTGCGATGCTGAAAGA R: TCGCCAGTCGAAAGTCGAAA	333	56	This study
*qnrC*	F: GCGAATTTCCAAGGGGCAAA R: AGCTGCTCTTGTTGCCATGA	515	56	This study
*qnrD*	F: CGAGATCAATTTACGGGGAATAG R:ACACCTAAACTCTCAACAAGCTGAA	596	56	(68)
*qnrE*	F: TGCGATTTATCGATGGCGGA R: AAGTCGCTCCATCAACTGGG	420	54	This study
*qnrS*	F: CGACGTGCTAACTTGCGTGATA R: TACCCAGTGCTTCGAGAATCAG	538	64	(68)
*qnrVC*	F: ACTGGAAGGGTGCGATTTTT R: ATGAAGCGCCTCGAAGATTT	302	56	This study

### Antimicrobial susceptibility testing

The antimicrobial susceptibilities of *qnr*-positive *Salmonella* strains to 14 antibiotics from 9 antimicrobial classes commonly used in human and veterinary medicine were determined by the broth microdilution method according to Clinical and Laboratory Standards Institute guidelines M100 and VET 08, and the European Committee on Antimicrobial Susceptibility Testing guidelines (http://mic.eucast.org/Eucast2). The selected antimicrobial drugs included ampicillin (AMP), ceftazidime (CEF), ceftriaxone (CRO), cefepime (FEP), cefoxitin (FOX), tetracycline (TET), trimethoprim-sulfamethoxazole (SXT), florfenicol (FFC), gentamicin (GEN), enrofloxacin (ENR), ofloxacin (OFL), levofloxacin (LVF), colistin (CL), and fosfomycin (FOS). *E. coli* ATCC25922 was used as quality control. Strains resistant to three or more antimicrobial classes were considered as MDR.

### Conjugation transfer experiment

Conjugation assays were performed using the filter mating method as described previously (69), using azide-resistant *E. coli* J53 as the recipient strain. Briefly, *qnr*-positive *Salmonella* strains (donor bacteria) and *E. coli* J53 (recipient bacteria) were mixed at a ratio of 1:3 and cultured on antibiotic-free LB agar plates attached with a 0.22 µm filter membrane overnight. After washing the bacterial lawn with 1 mL antibiotic-free LB broth and 10-fold gradient dilution, the bacterial solution was spread on LB agar plates containing 150 µg/mL azide and 0.1 µg/mL enrofloxacin (selecting transconjugants), and LB agar plates containing 150 µg/mL azide (selecting recipients), respectively. The conjugation transfer efficiency = the number of transconjugants/the number of recipients. The plasmid types of the transconjugants were identified by PCR (70).

### Whole genome sequencing

Based on different resistance phenotypes and the isolation region of strains, 47 non-replicated strains, including 43 *qnrS*-positive and 4 *qnrB*-positive strains, were selected and further subjected to WGS.

Genomic DNA was extracted from strains using the Genomic DNA Purification Kit (Tiangen, Beijing, China). Qubit 2.0 was used for preliminary quantification, Agilent 5400 was used to detect the insertion size of the library diluted to 2 ng/µL, and q-PCR was utilized to accurately quantify the effective concentration of the library and ensure the quality of the library. Trimmatic (v0.36) software was used to control the data quality of the qualified library. Spades (v3.13.0) software was used to assemble and statistically analyze the sequencing data of each strain after quality control, and finally, the scaffold sequence greater than 500 bp was chosen for further analysis.

### Bioinformatic analysis

The SeqSero2 database (https://cge.food.dtu.dk/services/SeqSero/) was used to predict serotypes. The PubMLST database (https://pubmlst.org/mlst) was used to determine the ST based on MLST of seven genes (*aroC*, *dnaN*, *hemD*, *hisD*, *purE*, *sucA*, and *thrA*) in *Salmonella*. A genomic comparison of 47 *qnr*-positive *Salmonella* strains was performed by Enterobase (https://cge.cbs.dtu.dk/services/cgMLSTFinder/) (71).

Resfinder 4.0 database (https://cge.cbs.dtu.dk/services/ResFinder/) with default parameters was used to annotate antimicrobial resistance genes (72). A database of heavy metal and disinfectant resistance genes for BacMet was created in abricate software (https://github.com/tseemann/abricate/) and parameters of ≥90% identity and ≥90% coverage were set to predict the presence or absence of heavy metal and disinfectant resistance genes (73). PlasmidFinder with default parameters (http://www.genomicepidemiology.org/) was used to identify plasmid replicons (74). The RAST website (https://rast.nmpdr.org/) was used to annotate contigs containing the *qnr* gene. The VFDB database (http://www.mgc.ac.cn/VFs/) was used for virulence gene annotation (75).

### Statistical analyses

The Pearson's correlation (*r*) between conjugation-positive strains and plasmids among 47 *qnr*-positive *Salmonella* strains was measured by SPSS (Version 22). Conjugation-positive strain and plasmid presence received scores of 1, whereas conjugation-negative strain and the absence of plasmid received scores of 0. Binary data (0/1) were imported into SPSS. The correlation was considered strong if the *r* ≥ 0.6, moderate if the *r* value was between 0.4 and 0.6, and weak if *r* < 0.4 (76).

## Supplementary Material

Reviewer comments

## Data Availability

The FASTQ reads from qnr-positive Salmonella strains selected in this study were deposited in National Center for Biotechnology Information and assigned under SRA accession no. PRJNA850515.

## SUPPLEMENTAL MATERIAL

OPEN PEER REVIEW reviewer-comments.pdf An accounting of the reviewer comments and feedback.

ASM does not own the copyrights to Supplemental Material that may be linked to, or accessed through, an article. The authors have granted ASM a non-exclusive, world-wide license to publish the Supplemental Material files. Please contact the corresponding author directly for reuse.
